# A Comparative Analysis of Residential Energy Consumption in Urban and Rural China: Determinants and Regional Disparities

**DOI:** 10.3390/ijerph15112507

**Published:** 2018-11-09

**Authors:** Feng Dong, Bolin Yu, Yifei Hua, Shuaiqing Zhang, Yue Wang

**Affiliations:** School of Management, China University of Mining and Technology, Xuzhou 221116, China; cumtdf@cumt.edu.cn (F.D.); huamichelle@foxmail.com (Y.H.); qq61@tju.edu.cn (S.Z.); m18234567643@163.com (Y.W.)

**Keywords:** residential energy consumption, urban-rural comparison, regional disparities, SUR estimation, Shapley value decomposition

## Abstract

Residential energy consumption (REC) has become increasingly important in constructing an energy-saving and environment-friendly society in China. The main purpose of this study is to provide a more in-depth analysis of the determinants of REC from an urban-rural segregation perspective, and quantify the contributions of individual determinants to the regional disparities of REC. Based on the extended STIRPAT (the stochastic impacts by regression on population, affluence, and technology) model, seemingly unrelated regression (SUR) estimation is employed to examine the impacts of various determinants of urban REC per capita (URECP) and rural REC per capita (RRECP) in a sample of China’s 30 provinces over the period 2007–2016. Then, following the results of SUR, this paper tries to explore the reasons for interprovincial disparities of URECP and RRECP by using the Shapley value decomposition. The empirical results show that income level and heating lead to an increase in URECP, while other factors, including the share of natural gas, average temperature, child dependency ratio and gross dependency ratio, significantly decrease URECP. In terms of RRECP, it is shown that old-age dependency ratio, income level and the share of coal consumption positively influence RRECP, while average temperature has a negative effect on RRECP. Specially, the effect of gross dependency ratio on RRECP is positive, indicating the non-working-age population causes more energy use than the working-age population in rural areas. According to the Shapley decomposition, rather than social-economic variables, climate and heating factors contribute the most to the interprovincial differences in URECP. Furthermore, it is found that income level is the most important factor accounting for inter-provincial differences in RRECP. The findings of this research are of great interest, not only to scholars in REC-related fields, but also to decision makers.

## 1. Introduction

China has been the world’s largest-growing energy market since 2000 [1], and it surpassed the United States as the largest energy consumer in 2009 [2] and carbon emitter in 2007 [3,4]. With the development of China’s economy and continuous improvements in residents’ income and living standards, residential energy consumption (REC) has also grown rapidly. REC in China has increased from 32,891.11 Mt coal equivalent in 2007 to 54,208.66 Mt coal equivalent in 2016, with annual average growth of 5.7%. Moreover, REC accounted for 12.8% of China’s final energy consumption in 2016 [5], becoming the second largest energy consumer following the industry sector. However, the proportion of REC in China is far smaller than that in developed countries, which is typically more than 20% [6] (such as the case of France, where the share is 25% [7]). The individual resident is the smallest and most basic energy consumer, and plays an important part in promoting energy conservation and emission reduction. Globally, it is expected that REC per capita will rise at an annual growth rate of 0.94% before 2040 [8]. REC per capita in China is only 30.2%, 37.7% and 68.4% of that in the U.S., U.K. and Japan, respectively [9]. Hence, with the continued growth of China’s economy and the acceleration of urbanization, there is large potential for increases in REC and its share in total final energy consumption. More importantly, the increase in REC has become the main cause of the rise in energy demand and carbon emissions. As the most populous country in the world, China will be faced with more serious challenges triggered by the expected increase in REC compared with developed countries. Specially, some countries, including those of eastern Asia, should make the largest efforts to decrease REC [10]. In China, the residential sector plays an important role in achieving energy conservation and emissions reduction targets as it has a significant energy saving potential.

Since economic reform in 1978, China has entered a stage of rapid urbanization and profound transformation. China’s population urbanization was 57.35% in 2016, but remains very low in contrast to industrialized economies (which are typically more than 75%) [3]. It is expected that the urbanization rate will increase to around 60% in 2020 [11] and 77.3% in 2050 [12]. With the migration of rural residents to cities or towns, the improvement of their living conditions may lead to more demand for energy services [13]. There is evidence to show that the transformation of a rural resident into an urban resident during the urbanization process will lead to an increase in energy consumption of 1085.26 kg coal equivalent (kgce) [14]. More specifically, urban REC (UREC) is larger than rural REC (RREC) in terms of both total and per capita levels. In addition, the growth rate of RREC is larger than that of UREC, at both the aggregate and per capita levels. It has been found that the gap between urban REC per capita (URECP) and rural REC per capita (RRECP) narrowed during 2007–2016 [5]. In detail, URECP was 395.40 kgce in 2016, almost the same as RRECP (390.31 kgce). This may largely result from the changes in resident lifestyle and energy consumption patterns in rural China. On the one hand, the development of the rural economy improves residents’ living standards. On the other hand, with income increasing, an increasing number of household appliances require more commercial energy. In fact, urban energy has chronically been the focus of China’s national commodity energy system, from which rural energy has long been excluded [15]. There are significant differences in income levels, consumption habits and lifestyles between urban residents and rural residents. Accordingly, previous studies have suggested the determining factors of REC usually differ between urban areas and rural areas [16,17]. Matos et al. [18] explore the factors accounting for the differences in water and energy consumption between urban and rural areas. Therefore, the urban-rural dual structure cannot be ignored when studying REC-related issues in China.

Another important feature of REC in China is that URECP and RRECP display significant regional differences among 30 provinces. Unfortunately, there is not sufficient attention paid to this important issue in the existing literature. In 2016, five provinces, including Heilongjiang, Liaoning, Ningxia, Jilin and Shaanxi, showed higher URECP and lower RRECP nationwide. Specially, Beijing had the maximum RRECP of 545.7 kg per person, while the lowest RRECP was recorded in Guangxi with a value of 107.3 kg per person. In addition, URECP was the lowest in Yunnan (127.6 kg per person) and a slightly higher level of URECP was reported by Jiangxi (148.1 kg per person) and Fujian (158.8 kg per person). However, Xinjiang had the largest URECP of 744.7 kg per person, which was almost six times larger than that of Yunnan. Therefore, designing energy strategies requires in-depth understanding of interprovincial inequalities of both URECP and RRECP.

China is currently faced with profound economic and social transformations, and is at a crossroad with rapid urbanization, an aging population, a decreasing economic growth rate and an energy transition. In this context, three research strands regarding REC have emerged. The first strand relates to understanding the urban-rural characteristics of REC in China. This study compares various determinants with respect to their effects on URECP and RRECP. The second strand of research focuses on population age structure, energy transition and income, which are important factors influencing URECP and RRECP. Exploring these driving forces is crucial in decreasing REC. The third strand of research concentrates on the issue of why URECP or RRECP varies across China’s provinces. In other words, what contributes to the disparities of URECP or RRECP among different provinces? In reality, little attention, however, has been paid to the above-mentioned issues in previous studies, which motivates the present study. Investigating these research questions could provide insightful support in achieving energy conservation targets and urban-rural coordinated development. To do so, we used province-level panel data for the period 2007–2016 and employed the seemingly unrelated regression (SUR) to study the effects of different influencing factors on URECP and RRECP. Then, the Shapley value decomposition was performed to analyze the reasons for interprovincial disparities of URECP and RRECP, which are quantitatively attributed to the contributions of individual determinants.

**Figure 1:**
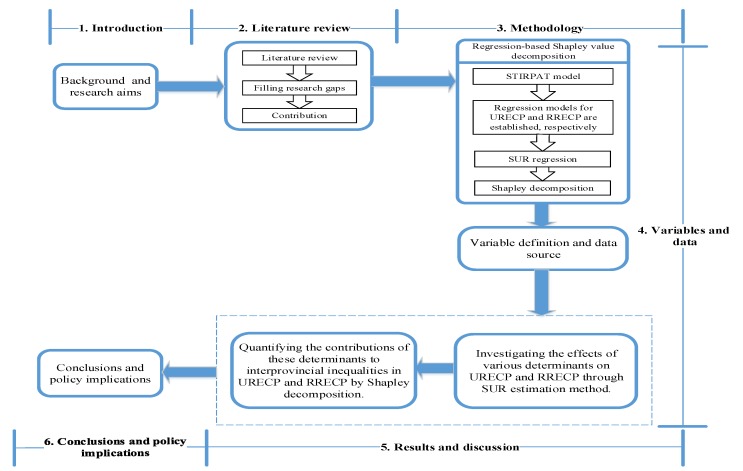
The framework of this study. STIRPAT: the stochastic impacts by regression on population, affluence, and technology; URECP: urban residential energy consumption per capita; RRECP: rural residential energy consumption per capita; SUR: seemingly unrelated regression.

To the best of our knowledge, the regression-based Shapley value decomposition has not been applied to REC-related studies. Through the combined SUR and Shapley decomposition analysis, this paper identifies the effects of various influencing factors on URECP and RRECP, and quantifies the contributions of individual determinants to interprovincial inequalities in URECP and RRECP. The integrated research framework is shown in Figure 1.

The rest of this paper is arranged as follows. Section 2 presents the literature review. Section 3 describes the methodology utilized in this paper. Section 4 summarizes all variables and presents the data sources. Section 5 provides the results and corresponding discussion. The final section concludes the research and offers some policy implications.

## 2. Literature Review

The increase in REC not only places pressure on energy security, but also leads to environmental issues [19], such as air pollutant emissions from household coal combustion [20]. As the largest developing country in the world, China has long regarded economic growth as its priority, which has been accompanied by considerable energy consumption. China’s policy makers have focused on energy-related issues in industrial production for a long time. However, with the rapid growth of REC in recent years, REC-related issues should also attract extensive attention and deserve insightful research. Analysis of the influencing factors and regional disparities of REC is of great practical significance in reducing carbon emissions, as well as in building an energy-saving and environment-friendly society.

There is growing concern over REC, and the driving forces of REC have generated considerable research interest. Many factors influence REC, such as income [21,22,23], price [17], age [24], population [25], energy mix [15], and expenditure structure [17,26]. Zhao et al. [26] employ the Logarithmic Mean Divisia Index (LMDI) method to study the effects of price, income, population, expenditure structure on UREC. Han and Wu [15] investigate the effects of energy structure transition, per capita income, price, juvenile dependency ratio, old-age dependency ratio and education level on RRECP.

Recently, China abolished the one-child policy and implemented a two-child policy as insurance against the approaching aging society. China’s population has chronically been characterized by a “smaller child population” and “aging of the population”. This is due to the implementation of the one-child policy and the decline in the population mortality rate because of the improvements in living standards and medical technology. China provides a typical data sample for studying the impact of changing population age structure on energy use. Accordingly, scholars have focused on the relationship between demographic factors and REC. There are significant differences in the energy consumption behaviors between the elderly and the young because an individual’s energy consumption changes distinctly within the life cycle [27,28]. Liddle [29] investigates the impacts of four key age groups on residential electricity consumption. The results specify the youngest and oldest cohorts have positive coefficients, while the coefficient of the middle cohort is opposite. Yamasaki and Tominaga [30] suggest that the evolution of an aged society would significantly influence REC. Using an overlapping generations model, Garau et al. [31] find that population aging has a negative effect on energy consumption, although it results in consumption of energy-intensive goods and services. Obviously, an aging society is unfavorable for economic development in the long term because of the loss of the demographic dividend, but there is evidence to indicate an aging society might objectively alleviate China’s environmental pressures to some extent [24].

Given the rapid urbanization process that transfers rural residents to urban areas, urbanization may also be an important factor influencing REC [25,32]. For example, Fan et al. [9] find urbanization may lead to an increase in REC, and contribute to decreasing coal consumption and increasing oil consumption. However, there is evidence that energy/electricity consumption results in urbanization [33]. Since the working-age population and non-working-age population display distinct heterogeneity in REC, it is clear that analyzing the compositional change of the population, especially the age composition and the distribution of people in urban and rural areas, is very important for understanding future needs and the potential for energy saving and emission reduction in residential sector. In addition to the above-mentioned socio-economic variables, climate is also known to be an important determinant of REC through influencing heating and cooling [17,34,35,36].

Apart from great differences in REC between urban and rural residents due to the existence of the urban-rural dual structure, the regional differences among different provinces should also attract scholars’ attention given China’s vast territory. The analysis of REC requires a more in-depth research from an urban-rural perspective covering a longer time period, as well as considering different development levels among regions. To the best of our knowledge, there are few studies focusing on the heterogeneity in REC across China’s provinces. For example, Herrerias et al. [37] examine the convergence patterns of REC in urban and rural China. Wang and Shi [38] compare URECP (including direct and indirect energy use) among different provinces, and they conclude energy use in inland areas is lower than that in coastal areas. However, these studies fail to find the causes of these differences and ways to diminish them, which is a research gap to be filled. In fact, there are distinct differences in economic development, technology, living habits and resource endowment among different regions [39]. REC also shows considerable heterogeneity and should similarly attract our attention. It would be very challenging to design energy strategies for different regions given these differences. Thus, it is necessary to provide an in-depth understanding of inter-provincial disparities of REC.

In this study, with consideration of urban-rural segregation, we try to identify the factors governing REC by the comparison of determinants of URECP and RRECP. Furthermore, this paper examines the heterogeneity in URECP and RRECP across different provinces, aiming at understanding the interprovincial disparities of URECP and RRECP and providing practical solutions for diminishing these differences. In doing so, the regression-based Shapley value decomposition, primarily developed in income inequality research [40,41], is performed in this paper. This approach can provide a string of interesting features when addressing Chinese data. First, according to the results of SUR estimation with province-level panel data for 2007–2016 in urban and rural China, we find that different factors have different effects on REC depending on rural or urban residents considered. Second, using Shapley value decomposition, we explicitly take into account the heterogeneity of URECP and RRECP across regions and over time. This aspect should be assigned considerable importance because of the significant changes involving China’s energy reform and economic development path, which lead to the well-known unbalanced nature of development across Chinese regions [39].

This paper contributes to the existing literature as follows. (1) With regard to REC, most studies investigate REC at national level with no regard for the differences between urban and rural areas. In this study the urban-rural dual structure is taken into consideration by respectively using urban and rural samples. (2) Although few studies have presented a comparison analysis of REC-related indicators between urban and rural areas [17,42], they all ignore the fact that a province’s non-observed factors may influence both UREC and RREC. When setting URECP and RRECP as explained variables, the disturbance terms of two equations may be correlated with each other. Based on the extended STIRPAT (the stochastic impacts by regression on population, affluence, and technology) model, this study employs the seemingly unrelated regression (SUR) method to investigate URECP and RRECP considering the connection between two disturbance terms, so as to improve the system estimation efficiency. (3) This research investigates the effects of demographic structure, per capita consumption expenditure, and energy mix on URECP and RRECP, together with the impacts of other determinants comprising climate and heating. More specifically, special attention is paid to the effects of three variables (i.e., gross, child and old-age dependency ratios) representative of the population age structure on URECP and RRECP. (4) To our knowledge, little study has focused on the regional disparities of REC among Chinese regions [37], and found ways to narrow these differences. The present study performs the Shapley value decomposition to identify the contributions of individual determinants to regional differences in URECP or RRECP, thereby proposing differentiated residential energy conservation policies. Our results provide new evidence of spatial heterogeneity among China’s provinces in terms of URECP and RRECP. It is believed that the methodology in the research has great significance particularly in obtaining an in-depth understanding of energy use in the REC sector. This study provides a good research framework for the next stages of REC-related research. The results of this paper not only extend the existing literature but also are of great importance to energy modelers and decision makers.

## 3. Methodology

### 3.1. Econometrics Model

Dietz and Rosa [43] propose the STIRPAT model, which is a stochastic form of the IPAT model (I=PAT, where *I*, *P*, *A* and *T* denote the impact per unit of economic activity, population effect, affluence effect and technology effect, respectively) [44]. The STIRPAT model is the basic analytical tool to study the non-monotonic or non-proportional impacts of driving forces of human activities on environmental pressure. The method of combining the STIRPAT model with econometrics is widely used in energy-related research. Compared with LMDI or the Tobit model, the STIRPAT model can not only treat coefficients as estimated parameters, but also disaggregate the impact factors into many proxy variables; in other words, *P*, *A* and *T* are dividable. Furthermore, other important influencing factors can be incorporated into the STIRPAT model [45]. The STIRPAT model can be expressed as follows:

(1) $$\;I=a\times P^{b}\times A^{c}\times T^{d}\times \varepsilon \;$$

where ε is the error term; *a* is a constant term; *b*, *c* and *d* denote the estimated exponents of *P*, *A* and *T*, respectively. Applying the natural logarithms to both sides of Equation (1) will yield:

(2) LnI=Lna+bLnP+cLnA+dLnT+ε. 

In this study, the affluence effect *A* is represented by per capita consumption expenditure, which reflects the changes in residents’ income level. In comparison to population size, the dependency ratio is the preferred indicator to denote population effect, *P*, which can reveal the structural changes of the population. The technology effect, *T*, is denoted by energy consumption structure. In addition, some variables highly related to URECP and RRECP are also considered in the model. Finally, Equation (2) can be extended as shown in the following equations:

(3) $$\;LnURECP_{it}=\beta_{0}+\beta_{1}LnCHILD_{it}+\beta_{2}LnOLD_{it}+\beta_{3}LnPCE_{it}+\beta_{4}SNG_{it}\;+\beta_{5}HEAT_{it}+\beta_{6}LnTEMP_{it}+\delta_{i}+\varepsilon_{it}.\;$$

(4) $$LnRRECP_{it}=\beta_{0}+\beta_{1}LnCHILD_{it}+\beta_{2}LnOLD_{it}+\beta_{3}LnPCE_{it}+\beta_{4}SC_{it}.\;\;+\beta_{5}LnTEMP_{it}+\delta_{i}+\varepsilon_{it}.\;$$

Equations (3) and (4) are regression models for URECP and RRECP, respectively, where i. denotes the i. th province and t. represents the tth year. In this study, REC is defined in per capita terms, which is more comparable in contrast to aggregate indicators. CHILD and OLD are respectively the child and old-age dependency ratios. PCE denotes per capita consumption expenditure. SNG represents the share of natural gas in UREC, and SC refers to the proportion of coal consumption in RREC, thereby eodying the energy transition trends in urban areas and rural areas, respectively. There was no natural gas consumption during 2007–2016 in most rural areas, such as rural Inner Mongolia, Guangdong and Heilongjiang. Specifically, natural gas consumption accounts for only 0.15% of total rural residential energy consumption [5]. Therefore, the energy mix in rural China is represented by SC with no regard for SNG. In fact, coal is mainly used for cooking and space heating in rural areas. By contrast, urban residents rarely burn coal for cooking, space heating or water heating. In most urban areas, such as urban Zhejiang, Hainan, and Tianjin, there is very little household coal consumption, thus, the effect of coal consumption on URECP is negligible. Meanwhile, China’s energy transition has promoted rapid growth in urban natural gas consumption. It is feasible to use SNG as a measure of the energy consumption structure in urban China. In addition to the above-mentioned socio-economic factors, some environmental and climate factors should also be taken into consideration, such as temperature and central heating. TEMP denotes average temperature of each provincial capital, and HEAT is a dummy variable indicating whether a province is enrolled in central heating. Given that central heating activities are concentrated in urban areas and there is no central heating in rural areas, HEAT is only introduced to Equation (3). All variables are expressed in the form of natural logarithms except HEAT, SNG and SC. $\varepsilon_{it}$ is a random error term irrelevant to time and region. $\delta_{i}$ is the regional non-observed effect, which specifies the impact of factors not changing with time. In other words, the persistent regional differences among provinces, such as lifestyle, consumption habits, resource endowment and economic aggregation may play an important role in influencing REC. Thus, we intend to consider regional fixed effects in the econometric models by introducing three regional dummy variables considering the significant regional differences between Eastern, Central, and Western China [4], so as to capture the heterogeneity across different regions.

According to microeconomic theory, price and income level are the dominant factors affecting commodity demand. Nevertheless, energy prices in China are largely influenced by the government. Thus, the marketization of energy prices is low, and the existing energy price mechanism cannot effectively reflect market reality. In fact, there is no uniform indicator to represent price changes in energy resources. In some cases, the application of price indicator may generate abnormal and misleading results with respect to the relationship between energy prices and energy consumption. The consumer price index (CPI) can reflect the change of energy prices to a certain extent. In this paper, per capita consumption expenditure at 2000 constant prices (converted by CPI) is employed to represent residents’ income, and can better reflect the changes in residents’ living standards and eliminate the impact of price fluctuations on energy consumption. Therefore, instead of per capita annual income of residents, per capita consumption expenditure is utilized as a proxy indicator of the affluence level of residents, thereby allowing the study of income and price effects on REC [6,23].

### 3.2. Regression-Based Shapley Value Decomposition

Regression-based inequality decomposition was first proposed by Fields and Yoo [46] and Morduch and Sicular [47]. This approach merges regression analysis and inequality decomposition in an integrated framework, and does not require the variable of interest to be expressed as the sum or product of several decomposed effects; on the contrary, it can attribute inequality to the contribution of any significant regressor. However, the method is restricted to the regression function form and inequality measure. Wan [40,41] then developed a modified framework combining a regression model with the Shapley value decomposition of Shorrocks [48]. The new approach can effectively resolve the deficiencies of traditional regression-based inequality decomposition. The regression-based inequality decomposition was originally developed to study income inequality issues, however, in recent years, it also has been applied to energy-related research [39,49,50]. For example, Dong et al. [39] perform the regression-based Shapley value decomposition to study the inequalities in energy intensity and energy consumption per capita among China’s 30 provinces using the Gini coefficient, Theil index (GE1) and mean logarithmic deviation (GE0) as inequality measures. According to Shapley value decomposition, if a certain regressor $x_{1}$ is equally distributed among 30 provinces, it will make no contribution to the inequality of the variable of interest. This study avoids overlap with Dong et al. [39], which has clearly described the methodology of the Shapley value decomposition. Similar to Dong et al. [39], we apply Shapley decomposition to decompose interprovincial inequalities of URECP and RRECP into the contributions of their determinants.

## 4. Variables and Data

### 4.1. Estimation of URECP and RRECP

In this study, REC refers to energy consumption for private transport and housing, such as cooking, space heating, water heating, cooling, lighting and home appliances. This paper mainly focuses on residential commercial energy consumption in China based on end use. The residential commercial energy types in the China Energy Statistical Yearbook mainly include raw coal, other washed coal, briquette, coke, coke oven gas, other coal gas, gasoline, kerosene, diesel, liquefied petroleum gas, natural gas, heat and electricity, whose conversion factors from physical units to coal equivalent are shown in Table 1. UREC and RREC of each province are obtained by aggregating various types of energy [51]. Therefore, urban residential energy consumption of province i in year t is estimated by

(5) $${UREC}_{it}=\sum_{j=1}^{13}E_{itj}\times \mu_{j}$$

where $E_{itj}$ denotes urban residential energy consumption of the j fuel type, and $\mu_{j}$ is its conversion factor in Table 1. Following this method, we obtain the data for RREC. Accordingly, URECP is measured by the ratio of UREC divided by urban population, and RRECP is given through the similar calculation process.

### 4.2. Data Sources

SUR and Shapley value decomposition are conducted in a sample of China’s 30 provinces over the period 2007–2016. The definitions of all variables are presented in Table 2. In this paper, the data of REC are measured in kg of coal equivalent (kgce), and the explained variable is defined as the final energy consumption per resident. With the course of energy transition in urban and rural China, advanced commercial energy gradually replaces traditional commercial energy, which is accompanied by an increase in the share of natural gas in urban areas and the decline in the share of coal in rural areas. Therefore, it is feasible to use energy mix as proxy indicator of technology effect. The energy-related data are mainly collected from the China Energy Statistical Yearbook [5], data on dependency ratios are from the China Population & Employment Statistics Yearbook [52], the dummy variable for central heating is obtained from the Department of Construction of China, and other data are derived from the China Statistical Yearbook [53].

## 5. Results and Discussion

### 5.1. The Influencing Factors of URECP and RRECP

**Figure 2:**
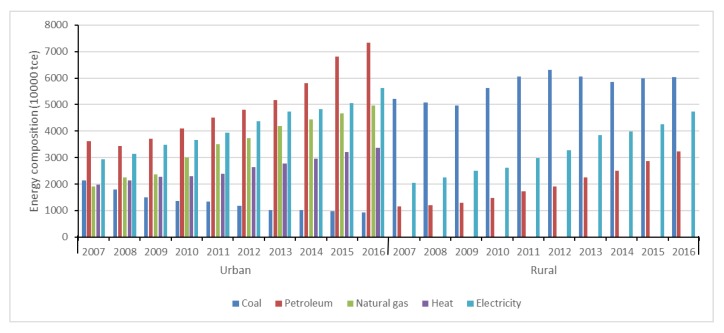
Composition of residential energy consumption in urban and rural China (t coal equivalent).

**Figure 3:**
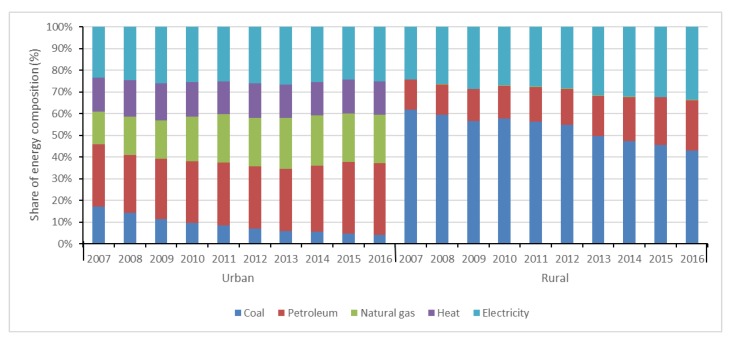
Changes of residential energy consumption structure in urban and rural China.

Compared with rural China, a more balanced energy consumption structure is found in urban China. As shown in Figure 2 and Figure 3, petroleum, natural gas, heat and electricity and their shares in UREC all showed increasing trends in terms of both absolute amount and relative proportion. However, coal presented a decreasing trend, and its share in UREC was only 4.15% in 2016. It is found that the urbanization process contributes to replacing coal with electricity in UREC [37]. That means energy consumption in the urban residential sector is in transition towards quality and cleaner energy, which reflects a low-carbon lifestyle and consumption mode in pursuit of a higher level of comfort, convenience and environmental protection.

With regard to RREC, the proportions of petroleum and electricity increased distinctly. By contrast, the proportions of natural gas and heat were almost 0 from 2007 to 2016, which reflects the trend of energy transition and changes in people’s living standards. Although the share of coal decreased during the analyzed period, residential commercial energy consumption continued to be dominated by coal, which indicates RREC mainly depends on the direct utilization of coal and will remain so for a long time. However, about 90.11% of the coal used in rural areas is raw coal of poor quality [15]. About 30% of coal is utilized for cooking and the thermal efficiency is low [54]. It is a significant challenge to improve the energy efficiency in rural regions and promote the shift of the energy mix towards quality energy sources, such as natural gas, electricity and oil products. Therefore, more efforts are required to optimize the energy mix in rural China.

The estimation results for URECP and RRECP are shown in Table 3 and Table 4, respectively, where Columns 2 and 4 report the results through a least square dummy variable model (LSDV) as baseline references, while Columns 3 and 5 present the results through SUR, a method of system estimation. Specially, the parameter estimates converge to the maximum likelihood results through the iteration algorithm (i.e., SUR_i). The results of the Breusch-Pagan Lagrange Multiplier test indicate the null hypothesis of no relation between the two disturbance terms of Equations (3) and (4) is rejected at the 1% significant level. That is to say, the SUR method is expected to be the preferred choice compared with traditional econometric methods. It is found that the estimated coefficients through LSDV are distinctly different from those through SUR_i. This is because SUR with system estimation is more efficient than LSDV with single equation, when taking the correlation between URECP and RRECP into consideration. Specially, given the estimated coefficients of child and old-age dependency ratios, this study employs the gross dependency ratio (represented by the ratio of non-working-age population to the working-age population) as a proxy indicator of population effect for comparison. Since there is a linear relationship between the child, old-age and gross dependency ratios, accordingly, the gross dependency ratio is included in Columns 4 and 5 without child and old-age dependency ratios.

Table 3 presents the effects of various determinants on URECP. The child dependency ratio has a negative effect on URECP and its estimated coefficients are significant at the 1% level. This finding conforms to the study of Brounen et al. [55]. Similarly, the increase in gross dependency ratio will lead to a decline in URECP. Energy demand for private transport is mainly related to the working-age population. Moreover, the ability of the working-age population to pay for household appliances and transport tools is higher than for the non-working-age population. However, the estimated coefficients of the old-age dependency ratio are insignificant, which means the effect of the old-age dependency ratio is relatively weak due to the hysteresis of age structure change and its influence. In the 1990s, the age structure of China’s population was skewed toward adulthood, and gradually began to transit towards the aged population. It is expected that the impact of the old-age dependency ratio on URECP will gradually rise along with population aging and increased life expectancy.

The elastic coefficient of PCE is significantly positive, which suggests per capita consumption expenditure contributes to increasing URECP. Our finding is in line with results of Yue and Long [6,23]. In addition, it should be noted that the elastic coefficient of PCE is less than unity, indicating the growth rate of URECP is smaller than that of PCE. This is because residential commercial energy is predominantly a necessity of life. According to the economic characteristics of necessities, when the income level is low, the income elasticity is relatively large. However, given diminishing marginal effects, the income elasticity will decrease as income increases. For urban residents with relatively high incomes and living standards, energy consumption will gradually maintain a stable level.

The estimated coefficients of SNG (denoted by the proportion of natural gas consumption in UREC) are negative, which specifies natural gas as a source of quality energy negatively influences URECP. This results from the fact that per unit natural gas can achieve higher energy efficiency than other energy types, especially coal. In recent years, the “coal to gas” project has been initiated by the government as an important strategy to handle energy and environmental issues. As a result, natural gas consumption exhibits a distinct upward trend in urban areas.

Central heating significantly contributes to increasing URECP. By contrast, average temperature has a negative effect on URECP, which is consistent with the study of Shen et al. [56]. In fact, heating energy consumption makes up a large share of UREC, and the rise in average temperature will lead to a decrease in energy consumption for heating. Similar estimated results have been addressed by previous research. For example, Miao [25] finds climatic characteristics and central heating activities are important factors determining UREC, while they have little effect on house-based residential CO_2_ emissions. In addition, Zhang [57] suggests that REC is highly related to heating degree days in China, Japan, Canada, and the United States.

In terms of RRECP, the impacts of various determinants on RRECP are reported in Table 4. Unlike urban residents, child dependency ratio has little influence on RRECP. By contrast, the old-age dependency ratio has a significant and positive effect on RRECP, which is in line with the finding of Yamasaki and Tominaga [30]. It indicates elderly households are more energy-intensive than other households. Furthermore, gross dependency ratio is also an important factor in increasing RRECP. Compared with the working-age population, the elderly population is characterized by a smaller number of household members, longer home occupancy, lower income levels, and spacious housing. Among these features, lower-income level may act as the only negative contributor to energy consumption, but the degree of its effect is limited. What’s more, the role of human migration should be considered when studying the impact of population structure on energy use in rural areas. Since the “Reform and Opening Up” policy in 1978, there are an increasing number of farmers migrating into urban areas who are mainly working-age people (i.e., peasant workers), which may be attributed to the urbanization process that transfers rural labor to urban areas. Hence, it is expected that the increase in gross dependency ratio will lead to the increase in RRECP.

It is found that PCE has a positive effect on RRECP, which is also the case with urban residents (Table 3). Specially, the elastic coefficient of PCE is larger than unity, which is different from the finding in Table 3. The growth rate of RRECP is faster than that of PCE for rural residents. This is mainly because with the improvement of rural living standards, residents become more amenity-conscious considering their energy needs, thereby resulting in a sharp increase in RRECP.

The estimated coefficients of SC are positive and significant at the 1% level, which specifies the increase in the proportion of coal consumption results in the increase in RRECP. This results from the fact that the energy utilization efficiency of coal is much lower than other energy types. Since the energy mix in rural areas has been long dominated by coal consumption, the rural energy consumption structure is still some way from being optimized.

The elastic coefficient of TEMP is negative and significant at the 1% level, which indicates average temperature negatively impacts RRECP. Increasing evidence suggests that climate plays an important role in influencing REC [17,34]. The findings of the present study reveal that temperature is a crucial determining factor of energy use for both urban and rural residents; that is to say, climate factors should be taken into consideration in future studies on residential energy use.

### 5.2. The Regional Disparities of URECP and RRECP

In the preceding section, the application of the SUR method is to analyze the effects of various factors on URECP and RRECP. Furthermore, an in-depth understanding of the regional disparities of URECP and RRECP among China’s provinces is helpful in formulating differentiated and practical energy conservation measures. Following the results obtained by SUR, the Shapley value decomposition provides a new perspective and can address the issue of why URECP/RRECP varies across different provinces in China.

From the perspective of heterogeneity analysis, it is important to find the reasons for regional differences in URECP and RRECP and narrow these differences, thereby reducing provincial residential energy consumption. From a statistical and economic point of view, significant variables would have decisive impacts on the inequalities in URECP and RRECP. The above-mentioned has paid special attention to the effects of three variables (i.e., gross, child and old-age dependency ratios) representative of population age structure. In Model (4) and Model (8), the estimated coefficients of the main explanatory variables are all significant. In addition, SUR estimation is more efficient than LSDV. As a result, Model (4) and Model (8) may be more appropriate than other models for the following decomposition analysis. In order to investigate the regional disparities of URECP and RRECP rather than LnURECP. and LnRRECP, we solve the regression equations according to Model (4) and Model (8), and obtain:

(6) $$\begin{array}{ll} \mathrm{URECP}= & \exp\left(3.963\right)\\ & *(-0.534*LnDEP+0.533*LnPCE-0.188*SNG+HEAT\\ & -0.563*LnTEMP+REG)*\exp\left(\delta_{i}\right)*\exp\left(\varepsilon_{it}\right) \end{array}$$

(7) $$\begin{array}{ll} \mathrm{RRECP}= & \exp\left(-8.381\right)\\ & *\exp(0.259*LnDEP+1.612*LnPCE+1.587*SC-0.563\\ & *LnTEMP+REG)*\exp\left(\delta_{i}\right)*\exp\left(\varepsilon_{it}\right) \end{array}$$

Since relative indices (i.e., Gini coefficient, GE1 and GE0) are used to gauge interprovincial inequalities in URECP and RRECP, the constant term contributes nothing to the inequalities. We estimate the contribution of regional fixed effects by integrating the estimated coefficients of regional dummy variables into a new variable REG. The final equations utilized for Shapley decomposition are given as:

(8) URECP=exp(−0.534∗LnDEP+0.533∗LnPCE−0.188∗SNG+HEAT−0.563∗LnTEMP+REG)

(9) RRECP=exp(0.259∗LnDEP+1.612∗LnPCE+1.587∗SC−0.563∗LnTEMP+REG)

**Figure 4:**
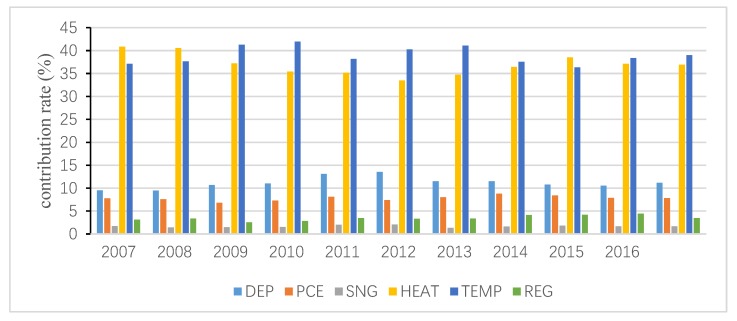
Decomposition result of urban residential energy consumption per capita (URECP) by Gini coefficient.

**Figure 5:**
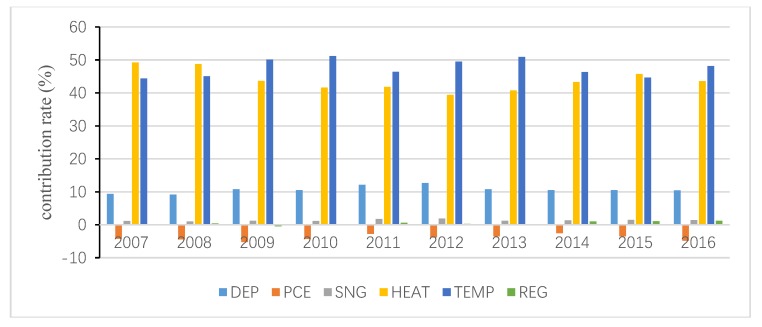
Decomposition result of URECP by GE1.

**Figure 6:**
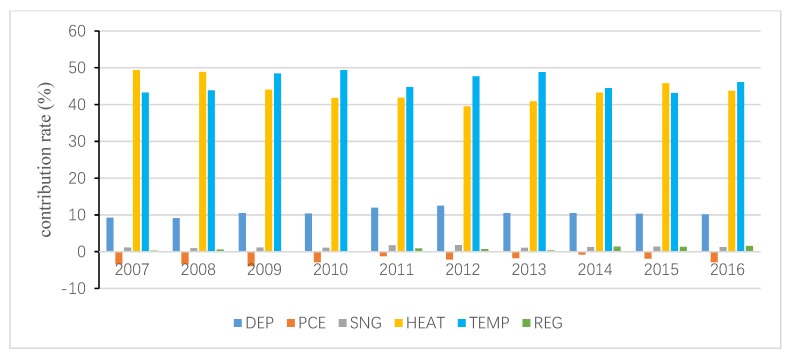
Decomposition result of URECP by GE0.

**Figure 7:**
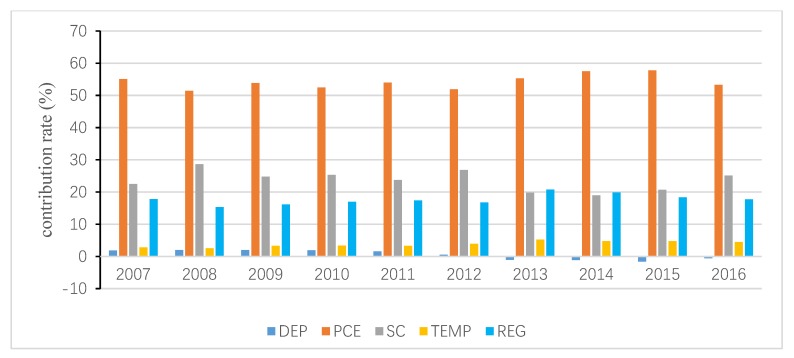
Decomposition result of rural residential energy consumption per capita (RRECP) by Gini coefficient.

**Figure 8:**
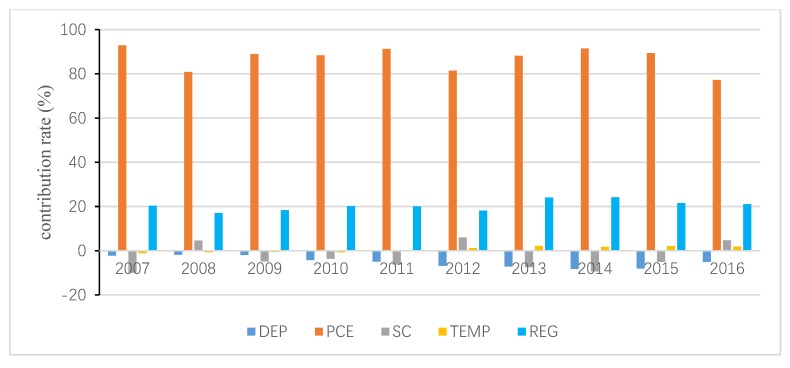
Decomposition result of RRECP by GE1.

**Figure 9:**
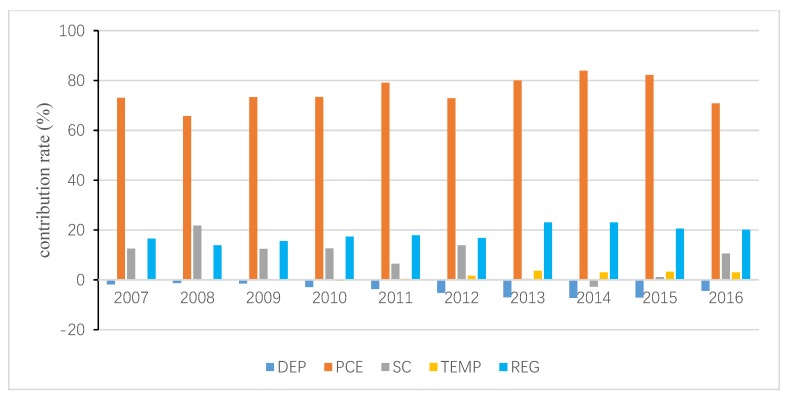
Decomposition result of RRECP by GE0.

The total inequality is attributed to the residual term and all independent variables, and the contribution of the residual term is obtained following Wan [40]. Then, the interprovincial inequality in URECP is attributable to interprovincial inequalities in DEP, PCE, SNG, HEAT, TEMP and REG. Similarly, the interprovincial inequality in RRECP is attributable to interprovincial inequalities in DEP, PCE, SC, TEMP and REG. The decomposition results are shown in Figure 4, Figure 5, Figure 6, Figure 7, Figure 8 and Figure 9, which indicate that the applications of different inequality measures lead to different results. The relative contributions of main determinants are not largely affected, thus, Figure 4 and Figure 7 can serve as the basic references. According to the Shapley decomposition, the independent variables in Equation (8) account for at least 78% of inequality in URECP during 2007–2016 when using the Gini coefficient as inequality measure; in other words, the residual term contributes little. By contrast, about 70–90% of inequality in RRECP can be explained by the explanatory variables in Equation (9) over the study period. The results suggest the model is successful in explaining the regional differences in URECP and RRECP, and Equations (8) and (9) are suitable to conduct the Shapley decomposition.

As shown in Figure 4, Figure 5 and Figure 6, followed by heating, temperature contributes the most to the inequality in URECP, which indicates that climatic characteristics and central heating activities rather than social-economic factors largely lead to the inequality in URECP among 30 provinces. This is because a large part of UREC is used for heating consumption, which is mainly related to regional temperature [56]. In addition, there is evidence that space heating makes up 50% of residential energy demand [58]. The third largest contributor to inequality in URECP is the gross dependency ratio, with an annual average contribution rate of 11% (see Figure 4), which indicates differences in the gross dependency ratio are an important factor in explaining interprovincial inequality in URECP. This is because the dependency ratio contributes to decreasing URECP following the results in Table 3. Furthermore, China’s population is unevenly distributed and there are significant regional differences in the gross dependency ratio. For example, URECP in Inner Mongolia is almost five times larger than that of Jiangxi, while Jiangxi’s dependency ratio is 1.5 times greater than that of Inner Mongolia. Following the gross dependency ratio, the annual average contribution rate of per capita consumption expenditure is 7.8%, which suggests that income also plays an important role in accounting for interprovincial differences in URECP. As for the regional fixed effect, its annual average contribution rate is only 3.5%. By contrast, interprovincial inequality in natural gas consumption makes the least contribution to interprovincial inequality in URECP, with an annual average contribution rate of 1.7%, indicating the gap of natural gas consumption is not a key factor resulting in regional disparities of URECP.

Figure 7, Figure 8 and Figure 9 present the contributions of individual determinants to interprovincial disparities of RRECP. Figure 7 specifies that per capita consumption expenditure is the most important factor accounting for inequality in RRECP: its annual average contribution rate even amounts to 87% according to Figure 8. This means RRECP is more easily influenced by income level than is URECP, which is highly consistent with the regression results in Table 3 and Table 4. Figure 7, Figure 8 and Figure 9 show different results with respect to the contribution rate of coal consumption because different inequality measures are sensitive to changes in high, medium or low levels of the variable of interest. With an annual average contribution rate of 23.7% (Figure 7), a larger share of coal consumption is an important factor accounting for higher RRECP in regions such as rural Hebei and Shanxi. Moreover, it should be noted that the regional fixed effect also plays an important role in explaining interprovincial inequality of RRECP. With an annual average contribution rate of 17.7% (see Figure 7), the regional fixed effect becomes increasingly prominent as its contribution rate gradually rises during the period 2007–2016. It is found that both gross dependency ratio and temperature make little contribution to inequality in RRECP, which indicates their impacts on RRECP are limited.

## 6. Conclusions and Policy Implications

Urban and rural areas are two different geographical spaces. During the process of urbanization, involving changes in residents’ income, production modes and lifestyles, energy consumption structures and energy consumption levels are largely different between urban and rural residents. Based on the extended STIRPAT model, this paper first employs the SUR method to investigate various influencing factors with regard to their effects on URECP and RRECP using panel data of 30 provinces during 2007–2016. Then, in order to analyze the inter-provincial differences in REC from the perspectives of URECP and RRECP, the Shapley decomposition is performed following the results of SUR. The main conclusions of this research are as follows. (1) The results of SUR indicate increasing income leads to an increase in URECP due to the promotion of purchasing power, and the share of natural gas consumption has a significant negative effect on URECP. Moreover, the child dependency ratio significantly and negatively influences URECP; however, the effect of the old-age dependency ratio is not significant. Further, investigation of the effect of the gross dependency ratio finds that the proportion of the non-working-age population living in urban areas negatively affects URECP. Additionally, climate and heating activities are also important factors influencing URECP. (2) The results of Shapley value decomposition specify that climate and heating activities are the largest contributors to inequality in URECP, and the third largest contribution is reported by the gross dependency ratio. The contributions of other factors are negligible over the study period. (3) The regression results show income also contributes to increasing RRECP, and coal consumption significantly influences RRECP. Furthermore, the old-age dependency ratio is significantly and positively correlated with RRECP, whereas the child dependency ratio does not influence RRECP substantially. In addition, the results show the proportion of non-working-age population living in rural areas positively influences RRECP. What’s more, climate is also an important factor determining the changes in RRECP. (4) According to the results of Shapley value decomposition, the gap in income is the most important reason accounting for regional differences in RRECP. However, the contributions of other factors are relatively small.

Designing residential energy conservation policies requires not only distinguishing urban-rural differences but also focusing on regional differences among different province. Based on the empirical results, policy implications are provided as follows.

In terms of URECP, climate and heating activities are the most important factors influencing URECP. First of all, there is a need to reduce household energy consumption in air conditioning and space heating, such as installing SolarWall^TM^ Systems. Given that central heating makes up a large part of UREC, it is indispensable to improve the heating efficiency and to reduce thermal loss of heating pipes effectively. Second, with increases in income level, it is necessary for every urban resident to enhance their awareness of energy conservation and promote energy-saving behaviors. From the perspective of final energy demand, since energy demand for private transport is an important part of UREC, the low carbon trip mode is encouraged for urban residents. Full advantage of public transport networks and shared bikes should be taken, thereby reducing energy demand for private transport. More importantly, the introduction and improvement of energy efficiency standards for households, thereby promoting the development of energy-saving technology and reducing energy demand for housing, such as from water heating, cooking and electrical appliances, should be undertaken. Third, there is still significant potential for future growth of residential natural gas consumption. It is imperative to popularize the use of natural gas and improve energy utilization efficiency. More specifically, three important aspects have emerged, i.e., reducing the cost of natural gas services, making the price mechanism of natural gas more market-oriented and enhancing the natural gas supply for residential use.

With respect to RRECP, the results show income is the main governing factor. Since China is still a developing country, it is of great significance to improve people’s living standards, especially for rural residents. Based on the actual conditions of rural China, a rise in income will lead to rapid increases in energy-intensive products, such as household electrical appliances and private cars. Therefore, it is of great importance to promote low-carbon consumption in rural areas. Specifically, policy makers should take actions such as providing subsidies or lowering taxes to encourage energy-saving electric appliances and new energy vehicles, including electric and hybrid vehicles with higher fuel efficiency. Second, the energy consumption structure should be optimized by fuel transition and advanced energy use technologies, thereby improving energy utilization efficiency and reducing energy consumption. In particular, the government should actively promote the implementation of “coal to gas” and “coal to electricity” projects. Third, in rural China, climate mainly influences RRECP by increasing coal consumption for heating in winter. It is thus necessary to promote the use of renewable energy, such as solar and geothermal energy. Fourth, in rural China, the non-working-age population can be encouraged to participate in physical exercise and outdoor activities, thereby reducing RRECP. In rural regions it is important to promote the construction of public facilities and infrastructure, such as sports fields, senior centers and nursing homes. Finally, persistent regional fixed factors cannot be ignored in policy making. Compared with the Eastern region, the Central and Western regions are characterized by backward economic development, low technology levels, and rich energy reserves, especially of coal. Thus, it is necessary in areas to popularize the use of electricity, gas, and solar energy, thereby reducing their dependence on coal.

## Figures and Tables

**Table 1:** Conversion factors from physical units to coal equivalent.

Energy	Conversion Factor	Energy	Conversion Factor
Raw coal	0.7143 kgce/kg	Kerosene	1.4714 kgce/kg
Other washed coal	0.2857 kgce/kg	Diesel	1.4571 kgce/kg
Briquette	0.7143 kgce/kg	Liquefied petroleum gas	1.7143 kgce/kg
Coke	0.9714 kgce/kg	Natural gas	1.1 kgce/cu·m
Coke oven gas	0.5714 kgce/cu·m	Heat	0.03412 kgce/mjoule
Other coal gas	0.1786 kgce/cu·m	Electricity	0.1229 kgce/kw·h
Gasoline	1.4714 kgce/kg		

Data source: National Bureau of Statistics of China [5]; Kgce: kg coal equivalent.

**Table 2:** Summary of variables in this study.

Variables	Definition	Source
Dependent variables		
URECP	Urban REC per capita	China Energy Statistical Yearbook and China Statistical Yearbook
RRECP	Rural REC per capita	China Energy Statistical Yearbook and China Statistical Yearbook
Independent variables		
CHILD	The ratio of the children population aged 14 and younger to the working-age population aged 15–64 in urban/rural areas	China Population & Employment Statistics Yearbook
OLD	The ratio of the elderly population aged 65 and older to the working-age population aged 15–64 in urban/rural areas	China Population & Employment Statistics Yearbook
DEP	The ratio of non-working-age population to the working-age population in urban/rural areas	China Population & Employment Statistics Yearbook
SNG	Share of natural gas in UREC	China Energy Statistical Yearbook
SC	Share of coal in RREC	China Energy Statistical Yearbook
PCE	Per capita consumption expenditure of urban/rural residents	China Statistical Yearbook
TEMP	Average temperature of each province	China Statistical Yearbook
HEAT	Dummy variable for central heating (HEAT = 1, or 0)	Department of Construction of China

**Table 3:** Determinants of urban residential energy consumption per capita (URECP).

Variables	Model (1)	Model (2)	Model (3)	Model (4)
	LSDV	SUR_i	LSDV	SUR_i
LnCHILD	−0.455 ***	−0.482 ***	—	—
	(0.101)	(0.099)	—	—
LnOLD	−0.074	0.007	—	—
	(0.085)	(0.081)	—	—
LnDEP	—	—	−0.562 ***	−0.534 ***
	—	—	(0.156)	(0.152)
LnPCE	0.540 ***	0.413 ***	0.614 ***	0.533 ***
	(0.104)	(0.101)	(0.102)	(0.099)
SNG	−0.222 *	−0.231 *	−0.178 ^#^	−0.188 ^#^
	(0.123)	(0.118)	(0.122)	(0.118)
HEAT	0.459 ***	0.407 ***	0.452 ***	0.406 ***
	(0.048)	(0.046)	(0.048)	(0.047)
LnTEMP	−0.465 ***	−0.487 ***	−0.526 ***	−0.563 ***
	(0.071)	(0.069)	(0.068)	(0.067)
DIST2	−0.001	−0.047	−0.013	−0.051
	(0.050)	(0.049)	(0.050)	(0.049)
DIST3	0.178 ***	0.152 ***	0.135 ***	0.104 **
	(0.053)	(0.052)	(0.051)	(0.050)
Constant	3.279 ***	4.371 ***	3.216 ***	3.963 ***
	(1.133)	(1.099)	(1.221)	(1.189)
Observations	300	300	300	300
R-squared	0.743	0.739	0.736	0.734
Breusch-Pagan	—	20.690 ***	—	14.678 ***

Note: Standard errors in parentheses, *** *p* < 0.01, ** *p* < 0.05, * *p* < 0.1, ^#^ *p* < 0.15; SUR_i denotes SUR through iteration algorithm.

**Table 4:** Determinants of rural residential energy consumption per capita (RRECP).

Variables	Model (5)	Model (6)	Model (7)	Model (8)
	LSDV	SUR_i	LSDV	SUR_i
LnCHILD	0.058	0.055		—
	(0.104)	(0.101)		—
LnOLD	0.334 ***	0.325 ***		—
	(0.098)	(0.094)		—
LnDEP	—	—	0.237 ^#^	0.259 *
	—	—	(0.150)	(0.146)
LnPCE	1.521 ***	1.499 ***	1.625 ***	1.612 ***
	(0.105)	(0.102)	(0.094)	(0.092)
SC	1.610 ***	1.614 ***	1.575 ***	1.587 ***
	(0.101)	(0.097)	(0.101)	(0.098)
LnTEMP	−0.244 ***	−0.237 ***	−0.194 **	−0.197 ***
	(0.080)	(0.077)	(0.077)	(0.075)
DIST2	−0.331 ***	−0.340 ***	−0.306 ***	−0.317 ***
	(0.064)	(0.063)	(0.064)	(0.064)
DIST3	−0.147 **	−0.159 **	−0.137 *	−0.153 **
	(0.071)	(0.070)	(0.073)	(0.072)
Constant	−7.685 ***	−7.496 ***	−8.417 ***	−8.381 ***
	(1.013)	(0.986)	(1.000)	(0.978)
Observations	300	300	300	300
R-squared	0.688	0.688	0.678	0.678
Breusch-Pagan	—	20.690 ***	—	14.678 ***

Note: Standard errors in parentheses, *** *p* < 0.01, ** *p*< 0.05, * *p* < 0.1, ^#^ *p* < 0.15; SUR_i denotes SUR through iteration algorithm.
